# Effects of tea residues-fermented feed on production performance, egg quality, antioxidant capacity, caecal microbiota, and ammonia emissions of laying hens

**DOI:** 10.3389/fvets.2023.1195074

**Published:** 2023-06-22

**Authors:** Xianxin Chen, Xinhong Zhou, Shiyi Li, Huaidan Zhang, Zhenkun Liu

**Affiliations:** ^1^Leshan Academy of Agriculture Science, Leshan, Sichuan, China; ^2^College of Life Science and Engineering, Southwest University of Science and Technology, Mianyang, Sichuan, China; ^3^Department of Animal Science and Technology, Chongqing Three Gorges Vocational College, Wanzhou, China

**Keywords:** laying hen, eggs quality, ammonia emissions, intestinal microflora, tea residues-fermented feed

## Abstract

This study was to assess the effects of tea residues-fermented feed (TR-fermented feed) on production performance, egg quality, serum antioxidant capacity, caecal microbiota, and ammonia emissions of laying hens. A total of 1,296 Lohmann laying hens have randomly distributed four groups with six parallels and fed with diets TR-fermented feed at the rates of 0 (control), 1, 3, and 5%. The inclusion of 1% (TR)-fermented feed resulted in a significant increase in egg-laying rate and average egg weight of birds, and a reduction in the feed-to-egg ratio when compared to the control group (*p* < 0.05). The addition of 1 and 3% of (TR)-fermented feed significantly improved the Haugh unit of eggs (*p* < 0.05). The eggshell thickness was observed to increase by almost one-fold upon the inclusion of 3 and 5% (TR)-fermented feed in the basal diet (*p* < 0.05). The supplementation of 3% (TR)-fermented feed significantly increased the content of methionine, tyrosine, proline, essential amino acids (EAA), alpha linoleic acid (C18:3n6), docosanoic acid (C22:0), docosahexaenoic acid (C22:6n3), twenty-three carbonic acids (C23:0), ditetradecenoic acid (C24:1) and total omega-3 polyunsaturated fatty acids (∑ω-3 PUFA) in the eggs (*p* < 0.05). The addition of a certain amount of (TR)-fermented feed can enhance the activity of glutathione peroxidase (GSH-PX) and superoxide dismutase (SOD) in chicken serum, and reduce the level of malondialdehyde (MDA) (*p* < 0.05). The ammonia concentration in the hen house of laying hens in the treatment groups decreased significantly (*p* < 0.05). *Bacteroidetes* and *Firmicutes*, the main phyla in the cecal bacterial community, were differentially abundant in each group, comprising greater than 55 and 33%, respectively. Collectively, this research indicates that (TR)-fermented feed supplementation improves the performance of laying hens and reduces ammonia emissions and can be used in industry-scale layer production.

## 1. Introduction

Fermented feed is used in poultry production to improve performance, immunity, and intestinal health, and to reduce the use of antibiotics (1–3). It is because the microbial fermentation of feed can reduce or eliminate the contents of toxic and harmful substances in raw materials effectively, while increasing the feed ingredients nutritional value and improving feed quality, thereby promoting the health of poultry (4–6). Probiotics such as *Lactobacillus*, *Saccharomyces*, and *Bacillus subtilis* have been widely used in feed fermentation, but it has been shown that the amount of protease secreted by the strains through simple microbial fermentation alone is low and cannot meet the actual production demand (7). Bacterial and enzyme co-fermented feed refers to the addition of a certain amount of exogenous compound enzymes and probiotics for joint fermentation, thus degrading the anti-nutritional ingredients in the feed, regulating the bitterness of the feed, improving the palatability of the feed, compensating for the lack of enzyme production and poor enzymatic taste of a single microbial fermentation, promoting animal feeding, improving feed utilization and nutritional value (8–10).

Tea residues are the by-products formed after the deep processing of tea. Although tea residues are the by-products of tea after extraction, they still contain more tea polysaccharides, theanine, tea polyphenols and other bioactive substances, which have antibacterial, antioxidant and anti-cancer functions (11–15). The use of tea residues in animal products not only improves the production performance, antioxidant capacity, and immunity of animals, but also avoids the pollution of the environment when tea residues are discarded in large quantities (16, 17). Adding tea powder or tea polyphenols to the feed can enhance the immunity and intestinal health of laying hens, increase their egg production rate, reduce cholesterol content in eggs, and improve egg quality (18–20). Solid-state fermentation of tea residues resulted in a significant increase in crude protein (CP), reducing sugar, and cellulose activity by 19.32, 39.5, and 33.3%, respectively, compared to their levels before fermentation (21). It was found that fermented tea pomace could improve the growth capacity and gut morphology of fattening pigs via improving nutrient utilization and intestinal digestive enzymes activities, and improve pork qualities via improving antioxidant properties of fattening pigs (17), and it has also been found that fermented tea residues can improve product performance and resistance to *A. hydrophila* in *Micropterus Salmoides* (16). However, in poultry farming, most of the research has been conducted on the use of tea residues as feed ingredients or functional additives. Therefore, the major objective of the present research was to use tea residues as part of a fermented feed by using co-fermentation with bacteria and enzymes. Tea pomace fermented feed has been supplemented in the diet of laying hens to investigate the effect on production capacity, egg quality, anti-oxidant capacity of serum, gut microbial composition, and ammonia emission from the poultry house.

## 2. Materials and methods

### 2.1. Diets

Table 1 displays the composition and nutrient levels of the basal diet, which was prepared in accordance with the Chinese chicken feeding standard (NY/T 33-2004) (22). Tea residues which include tea leaves and tea stalks were provided by Sichuan Baiyue Tea Industry. The probiotic used for fermentation contains *B. subtilis* ≥ 1.5 × 10^9^ CFU/g, *L. plantarum* ≥ 1.0 × 10^9^ CFU/g were provided by the Leshan Academy of Agricultural Sciences, the enzymes used for fermentation contains β-mannanase ≥50 U/g, β-glucanase ≥3,000 U/g, cellulase ≥1,000 U/g, and xylanase ≥500 U/g were purchased from market, and product standard No. Q/12JX 4450-2019. Our fermentation procedure is to crush the feed ingredients for fermented feed, weigh all the ingredients accurately according to the ratio in Table 2 and place them in fermentation bags containing a one-way breathing valve, mix them thoroughly and then place them at 36°C for 96 h.

**Table 1:** Composition and nutrient levels of the basal diet (air-dried basis, %).

Ingredients	Contents	Nutrient levels^2^	Contents
Corn	60	Digestive energy (MJ/kg)	11.27
Soybean meal	19	Crude protein	15.83
Wheat bran	6	Calcium	3.51
Corn protein powder	2.8	Available phosphorus	0.33
Soybean oil	1.8	Lysine	0.78
CaHPO_4_	1.25	Methionine	0.40
CaCO_3_	7.65		
Premix^1^	1.5		
Total	100		

1 The premix contained the following per kg: vitamins B_2_ (6 mg), B_1_ (4 mg), B_6_ (3 mg), E (20 IU), D_3_ (4,000 IU), A (7,000 IU), K_3_ (4 mg), niacin (20 mg), choline chloride (500 mg), folic acid (1 mg), biotin (0.2 mg), pantothenic acid (10 mg), Zn (80 mg), Se (0.3 mg),Cu (15 mg), Mn (100 mg), Fe (60 mg), and I (0.4 mg).

2 Nutrient levels were measured in values, except metabolic energy.

**Table 2:** Tea residues-fermented feed composition and nutrient levels (fresh base, %).

Ingredients	Contents	Nutrient levels	Contents
Corn	16.25	Water content	29.32
Soybean meal	11	Digestive energy (MJ/kg)	10.79
Corn germ meal	10	Crude protein	13.57
Spraying corn husks	10	Tea polyphenols	2.41
Tea residues	22	Calcium	2.5
CaHPO_4_	1.3	Available phosphorus	0.3
Limestone	6	Lysine	0.84
NaCl	0.25	Methionine	0.39
Water	23		
Methionine	0.2		
Total	100		

Nutrient levels were measured in values, except metabolic energy.

### 2.2. Animals

In this study, a total of 1,296 healthy Lohmann laying hens at 34 weeks of age were assigned randomly to 4 dietary groups (T0–T3), each with 6 parallels of 54 laying hens. Three chickens were raised in cages (47 cm × 45 cm × 44 cm). The chickens were hand-fed three times a day (06:30 am, 11:30 am, and 4:30 pm) for the duration of the experiment. The hens have access to free drinking water with 16 h of continuous light per day. T0 (control group) fed the basal diet, T1, T2 and T3 replaced 1, 3, and 5% of the basal diet with tea residues-fermented feed, respectively. The hens were acclimated to the experimental conditions for 7 days and the experiment lasted 6 weeks.

### 2.3. Sample collection

At the end of the trial, randomly collected 6 eggs per replicate, six chickens were selected randomly from each treatment (one per replicate) and fasted for 12 h before sampling. The blood was collected from the wing vein using a blood collection needle, and 5 mL of it was transferred to a vacuum tube. The tube was kept at room temperature to allow for serum separation, after which it was centrifuged at 3,000 × *g* for 30 min at 4°C. The serum was then stored at a temperature of −20°C. The cecum was collected in a sterilized centrifuge tube. Buffered peptone broth was used to homogenize each portion, which was then mixed with 10% sucrose and divided into aliquots. The aliquots were frozen at a temperature of −80°C (23).

### 2.4. Production performance

Throughout the trial, daily records were made of the number of feed intake, eggs produced, and total egg weight for each replicate. Subsequently, the egg-laying rate, average egg weight, and feed-to-egg ratio were determined for the periods of 1–22 days and 22–42 days.

Egg-laying rate (%) = (number of egg-laying/number of chickens) × 100

Average egg weight = total egg weight / number of eggs laid

Feed intake = feeding amount–surplus

Feed-to-egg ratio = feed intake/total eggs weight

### 2.5. Eggs quality

The collected eggs were tested using an automatic quality analyzer (TOUHOKU RHYTHM CO., LTD EMT-7300) for egg yolk color, albumen height and Haugh units. The eggshell thickness was measured using the YN-25L egg quality analyzer, which measured the eggshell at three different locations and took the average value as the eggshell thickness of the egg. To calculate the egg shape index, vernier calipers were used to measure the long and short diameters of the eggs.

Egg shape index = long diameter/short diameter

### 2.6. Determination of amino acids and fatty acids in eggs

Transfer the eggs to a clean small beaker, mix the egg yolk and egg white thoroughly, weigh 0.1 g of the sample and place it in a hydrolysis tube. Add 6 mol/L of concentrated hydrochloric acid to 10 mL, seal the tube, and place it in a constant temperature drying oven at 110°C for 24 h. After the digestion is complete, cool to room temperature, accurately pipette 1 mL of the filtrate, and place it in a rotary evaporator in a 50°C vacuum drying box. Then, add 2.0 mL of pH 2.2 sodium citrate buffer solution to the dried test tube, shake well, and filter the solution through a 0.22 um filter membrane. Place the supernatant in an amino acid analyzer (Hitachi, LA8080) for determination (24).

Take a uniform sample weighing 2 g and transfer it to a flask. Add around 100 mg of pyrogallic acid and a small quantity of zeolite. Next, pour in 2 mL of 95% ethanol and 10 mL of hydrochloric acid solution, and mix the contents thoroughly. The flask should then be placed in a water bath maintained at a temperature of 70°C ~ 80°C for 40 min to facilitate hydrolysis. Rewrite the following passage:Weigh 2 g of a uniform sample and place it in a flask. Add approximately 100 mg of pyrogallic acid and a few grains of zeolite. Then add 2 mL of 95% ethanol and 10 mL of hydrochloric acid solution, mix well. Place the flask in a 70°C ~ 80°C water bath for 40 min to hydrolyze. In a 250 mL flask, collect the ether layer extract obtained from the previous step. Repeat the process thrice to extract the hydrolyzate. Lastly, rinse the separating funnel with a combination of ether and petroleum ether and collect the rinsing liquid in the flask until a constant weight is achieved. The flasks were dried on a water bath and then dried in an oven for 2 h at 100°C. Thereafter, add 2 mL of 2% sodium hydroxide methanol solution and place it in an 85°C-water bath for 30 min. Next, 3 mL of a 14% methanolic solution of boron trifluoride was added and placed in a water bath for 30 min at 85°C. When the water bath has finished, allow the temperature to drop to room temperature. Addition of 1 mL of n-hexane to the tube was centrifuged, shaken, and extracted for 2 min. Allow the contents to remain an hour to allow stratification. Extract 100 μL of the upper clear liquid, dilute it to 1 mL using n-hexane, and filter it through a 0.45 μm filter membrane. Finally, use a gas chromatography mass spectrometer (Trace1310 ISQ, Thermo) to determine the results.

### 2.7. Serum antioxidant capacity

The total superoxide dismutase (SOD) and glutathione peroxidase (GSH-PX) activities and serum malondialdehyde (MDA) levels were measured by spectrophotometer or enzyme marker using kits from Nanjing Jiancheng Institute of Biological Engineering according to the manufacturer’s instructions (25).

### 2.8. Ammonia emissions

The same sites in the hen houses were selected for the experimental and control groups at equal distances from top to bottom. An inverted 6-cm-diameter, the 12-cm-high container was placed above a cage at 15:00 every day. After 2 h, the ammonia gas was detected with a Wandi GASTiger 2000 (26).

### 2.9. Intestinal microflora

The collected cecum contents were weighed 250 mg and the microbial DNA was extracted from the samples using the EZNA soil DNA kit (Omega Bio-Tek, Norcross, GA, United States) as per the manufacturer’s instructions. Using an ABI Gene Amp 9700 PCR thermocycler (ABI, CA), the primer pairs 338F (5′-ACTCCTACGGGAGGCAGCA-3′) and 806R (5′-GGACTACHVGGGTWTCTAAT-3′) were employed to amplify the hypervariable region V3−V4 of the bacterial 16S rRNA gene. The PCR reactions commenced with an initial denaturation at 98°C for 2 min. This was followed by 30 cycles of denaturation at 98°C for 30 s, annealing at 55°C for 30 s, and extension at 72°C for 60 s. The final extension was carried out at 72°C for 5 min. The AxyPrep DNA Gel Extraction Kit (Axygen Biosciences, Union City, CA) was utilized to extract and purify the amplicons as per the manufacturer’s instructions. The kit was effective in removing excess primer dimers and dNTPs from the sample. Equal amounts of purified amplicons were combined and subjected to paired-end sequencing (2*250 bp) using the Illumina MiSeq platform at Tsingke Biotechnology Co., Ltd., (27).

### 2.10. Statistical analysis

The experimental data were recorded in Microsoft Excel 2010 and the results were analyzed using SPSS 23 was used to perform one-way ANOVA (One-way ANOVA) on the experimental results, and the Duncan’s method was used for multiple comparisons, with *p* < 0.05 as a significant difference. Data are presented in the table as mean and combined SEM. The figure was drawn using GraphPad Prism6 (GraphPad Software, United States).

## 3. Results

### 3.1. Production performance

Table 3 displays the production performance of laying hens fed (TR)-fermented feed; no significant changes in the ADFI between the experimental groups were found (*p* > 0.05). During the period of 1–21 days, the Egg-laying rate and Average egg weight of the T1 group of broiler chickens were significantly higher than those of the T0 and T3 groups (*p* < 0.05). During the period of 22–42 days, the Egg-laying rate of the T1 group was significantly higher than that of the T0, T2, and T3 groups. Additionally, the Feed-to-egg ratio of the T1 group was significantly lower than that of the T3 group (*p* < 0.05). Throughout the entire period of 1–42 days, the Egg-laying rate of the T1 group was significantly higher than that of the T0, T2, and T3 groups. Moreover, the Average egg weight of the T1 group was significantly higher than that of the T0 group, and the Feed-to-egg ratio of the T1 group was significantly lower than that of the T0 and T3 groups (*p* < 0.05).

**Table 3:** The effects of adding (TR)-fermented feed on the production performance of laying hens.

Items	T0	T1	T2	T3	SEM	*p*-value
1–21 days
ADFI/g	108.77	108.59	107.73	109.43	1.30	0.982
Egg-laying rate/%	88.86^b^	91.90^a^	90.53^a^	87.70^b^	0.53	<0.05
Average egg weight/g	55.46^c^	56.83^a^	56.64^ab^	56.20^b^	0.17	<0.05
Feed-to-egg-ratio	2.22	2.08	2.10	2.23	0.03	0.267
22–42 days
ADFI/g	113.71	117.70	117.29	118.66	1.03	0.386
Egg-laying rate/%	88.04^b^	92.33^a^	89.17^b^	86.74^b^	0.73	<0.05
Average egg weight/g	56.80	57.78	57.77	57.78	0.24	0.441
Feed-to-egg-ratio	2.28^ab^	2.21^b^	2.28^ab^	2.37^a^	0.02	<0.05
1–42 days
ADFI/g	110.91	113.31	112.51	113.88	1.17	0.842
Egg-laying rate/%	88.45^c^	92.12^a^	90.11^b^	87.35^c^	0.45	<0.05
Average egg weight/g	56.11^b^	57.30^a^	57.22^ab^	56.99^ab^	0.20	<0.05
Feed-to-egg-ratio	2.27^a^	2.14^b^	2.23^ab^	2.29^a^	0.02	<0.05

Values in the same row without or with the same letter superscripts indicate no significant difference (*p* > 0.05). Values with different small letter superscripts indicate a significant difference (*p* < 0.05).

### 3.2. Eggs quality

Table 4 displays the egg quality after 42 days of feeding the hens (TR)-fermented feed. Between each group’s albumin height and Egg shape index, there was no discernible variation (*p* > 0.05). When compared to the T0 group, T1 and T2 group significantly enhanced the Haugh unit of eggs (*p* < 0.05). It is notable that the T2 and T3 group considerably and nearly multiplicatively enhanced the eggshell thickness of the eggs (*p* < 0.05).

**Table 4:** The effects of adding (TR)-fermented feed on the egg’s quality of laying hens.

Items	T0	T1	T2	T3	SEM	*p*-value
Egg shape index	1.35	1.29	1.31	1.31	0.01	0.421
Yolk color	6.72^ab^	6.86^a^	5.92^b^	6.54^ab^	0.14	<0.05
Albumen height/mm	6.00	6.70	7.28	6.32	0.36	0.651
Haugh unit	77.44^b^	87.18^a^	91.14^a^	78.74^b^	1.59	<0.05
Eggshell thickness/mm	0.44^b^	0.46^b^	0.70^a^	0.85^a^	0.04	<0.05

Values in the same row without or with the same letter superscripts indicate no significant difference (*p* > 0.05). Values with different small letter superscripts indicate a significant difference (p < 0.05).

### 3.3. Amino acid and fatty acid contents

As shown in Table 5, we detected a total of 16 amino acids in the eggs, and the addition of (TR)-fermented feed has increased the content of most amino acids in the eggs. Compared with the T0 group, the T2 group enhanced the content of Methionine, Tyrosine, Proline, and EAA in the eggs (*p* < 0.05). The fatty acid content in eggs after supplementation of (TR)-fermented feed is shown in Table 6. The content of C17:0 in eggs with T1 and T3 was higher than that in the control group (*p* < 0.05). The T2 group significantly increased the content of C18:3n6, C22:0, C22:6n3, C23:0, C24:1, and ∑ω-3 PUFA in the eggs (*p* < 0.05). The content of ∑SFA, ∑MUFA, ∑PUFA, and ∑ω-6 PUFA in eggs supplemented with (TR)-fermented feed was higher than that in the control group (*p* > 0.05).

**Table 5:** The effects of supplemented (TR)-fermented feed on the amino acid contents of eggs from laying hens.

Items^1^	T0	T1	T2	T3	SEM	*p*-value
Aspartic acid	10.13	10.25	11.49	10.63	0.26	0.270
Threonine	4.91	5.06	5.57	5.22	0.13	0.287
Serine	7.07	7.39	8.19	7.66	0.19	0.188
Glutamic acid	12.47	12.64	14.04	12.97	0.31	0.310
Glycine	3.39	3.43	3.80	3.54	0.09	0.353
Alanine	5.90	6.01	6.69	6.19	0.15	0.294
Valine	6.74	6.73	7.61	7.02	0.18	0.288
Methionine	2.32^b^	1.87^b^	3.72^a^	2.15^b^	0.26	<0.05
Isoleucine	5.28	5.27	6.07	5.63	0.15	0.167
Leucine	9.15	9.37	10.51	9.77	0.24	0.217
Tyrosine	3.60^b^	3.75^ab^	4.47^a^	3.92^ab^	0.13	<0.05
Phenylalanine	5.88	5.90	6.49	6.07	0.15	0.500
Lysine	7.27	7.45	8.29	7.62	0.19	0.257
Histidine	2.20	2.24	2.49	2.35	0.07	0.473
Arginine	6.31	6.46	7.26	6.84	0.17	0.207
Proline	3.12^b^	3.48^ab^	3.83^a^	3.51^ab^	0.11	<0.05
TAA	95.72	97.30	110.52	101.08	2.64	0.192
EAA	41.47^b^	41.59^b^	48.62^a^	43.75^ab^	1.25	<0.05
NEAA	45.96	47.22	52.53	48.57	1.20	0.250
DAA	41.37	41.98	46.98	43.31	1.08	0.269

Values in the same row without or with the same letter superscripts indicate no significant difference (*p* > 0.05). Values with different small letter superscripts indicate a significant difference (*p* < 0.05).

1 DAA, delicious amino acids; NEAA, Non-essential amino acids; EAA, essential amino acids; TAA, total amino acids.

**Table 6:** The effects of supplemented (TR)-fermented feed on fatty acid content in eggs of laying hens.

Items^1^	T0	T1	T2	T3	SEM	*p*-value
C10:0	3.19	2.94	3.01	2.99	0.15	0.956
C12:0	5.10	5.14	5.84	4.87	0.22	0.457
C14:0	206.80	192.89	220.22	199.91	11.08	0.871
C14:1	53.01	41.73	49.13	43.07	2.94	0.535
C15:0	26.43	27.46	30.11	30.39	1.30	0.683
C16:0	11924.07	12217.55	12606.35	11783.79	360.59	0.885
C17:0	44.09^b^	56.92^a^	53.25^ab^	55.60^a^	1.93	<0.05
C17:1	33.38	36.43	34.76	36.22	1.30	0.857
C18:0	4522.53	5121.36	5250.85	5051.98	162.78	0.443
C18:1n9c	14468.87	15958.63	15081.92	14734.77	434.38	0.688
C18:2n6c	7833.52	8899.73	8480.35	7893.23	240.34	0.369
C18:3n3	284.94	327.50	330.22	354.65	17.44	0.604
C18:3n6	90.30^b^	135.60^ab^	140.46^a^	112.95^ab^	8.13	<0.05
C20:0	15.39	14.15	12.30	15.53	0.73	0.406
C20:1	95.03	104.16	103.14	119.67	4.65	0.321
C20:2	93.71	99.02	105.52	112.26	3.95	0.408
C20:3n6	263.16	264.07	296.88	258.61	12.07	0.703
C20:4n6	1205.35	1360.21	1559.45	1230.45	77.09	0.375
C20:5n3	28.84	32.49	33.90	27.98	1.33	0.358
C21:0	2.92	2.50	2.76	3.23	0.14	0.367
C22:0	8.89^b^	11.17^ab^	13.71^a^	11.86^ab^	0.68	<0.05
C22:6n3	4175.01^b^	4928.54^ab^	6209.19^a^	5242.36^ab^	318.15	<0.05
C23:0	4.91b	6.32ab	9.78a	7.81ab	0.77	<0.05
C24:0	15.42	15.06	18.63	17.07	0.81	0.411
C24:1	13.67^b^	16.09^ab^	22.04^a^	9.23^ab^	1.84	<0.05
∑SFA	16779.74	17673.46	18226.80	17185.03	515.37	0.813
∑MUFA	14663.96	16157.04	15290.99	14942.96	437.22	0.693
∑PUFA	13974.86	16047.16	17155.97	15232.49	579.36	0.274
∑ω-3 PUFA	4488.79^b^	5288.53^ab^	6573.31^a^	5624.99^ab^	324.29	<0.05
∑ω-6 PUFA	9392.35	10659.61	10477.15	9495.24	308.23	0.363

Values in the same row without or with the same letter superscripts indicate no significant difference (*p* > 0.05). Values with different small letter superscripts indicate a significant difference (*p* < 0.05).

1 C10:0, Aconitic acid; C18:0, Stearic acid; C20:1, Gadoleic acid; C12:0, Lauric acid; C20:5n3, Eicosapentaenoic acid; C20:2, Eicosadienoic acid; C17:1, Heptadecanoic acid; C18:2n6c, Linoleic acid; C14:1, Myristylic acid; C22:6n3, Docosahexaenoic acid; C20:3n6, Dihomo-y-linolenic acid; C18:3n3, Linolelaidic acid; C15:0, Pentadecylic acid; C22:0, Docosanoic acid; C18:3n6, Alpha linoleic acid; C14:0, Myristic acid; C16:0, Palmitic acid; C17:0, Margaric acid; C18:1n9c, Oleic acid; C20:0, Arachidonic acid; C20:4n6, Arachidonic acid; C21:0, Undecanoic acid; C23:0, Twenty-three carbonic acid; C24:0, Ditetradecanoic acid; C24:1, Ditetradecenoic acid; ∑SFA, Total Saturated Fatty Acids; ∑MUFA, Total monounsaturated fatty acids; ∑PUFA, Total polyunsaturated fatty acids; ∑ω-3 PUFA = (C22:6n3 + C20:5n3+ C18:3n3); ∑ω-6 PUFA = (C20:4n6+ C18:3n6+ C20:3n6+ C18:2n6c).

### 3.4. Serum antioxidant capacity

**Figure 1:**
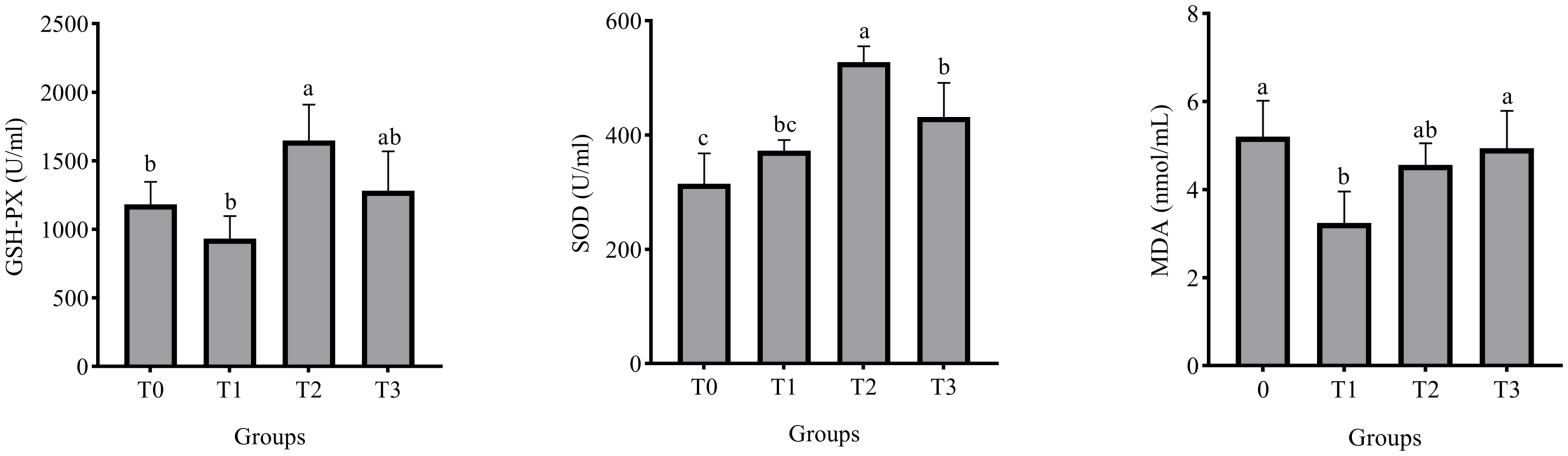
Effect of (TR)-fermented feed supplementation on the serum antioxidant capacity. MDA, malondialdehyde; SOD, superoxide dismutase; GSH- PX, glutathione peroxidase. Different letters indicate significant differences (*p* < 0.05).

Figure 1 displays the laying hens’ serum biochemical indicators. The addition of 3% (TR)-fermented feed substantially raised the GSH-PX and SOD activities of laying hens as compared to the control group (*p* < 0.05). There was a significant change with a 1% addition in the MDA content in the serum of laying hens compared to the control group (*p* < 0.05).

### 3.5. Ammonia emissions

**Figure 2:**
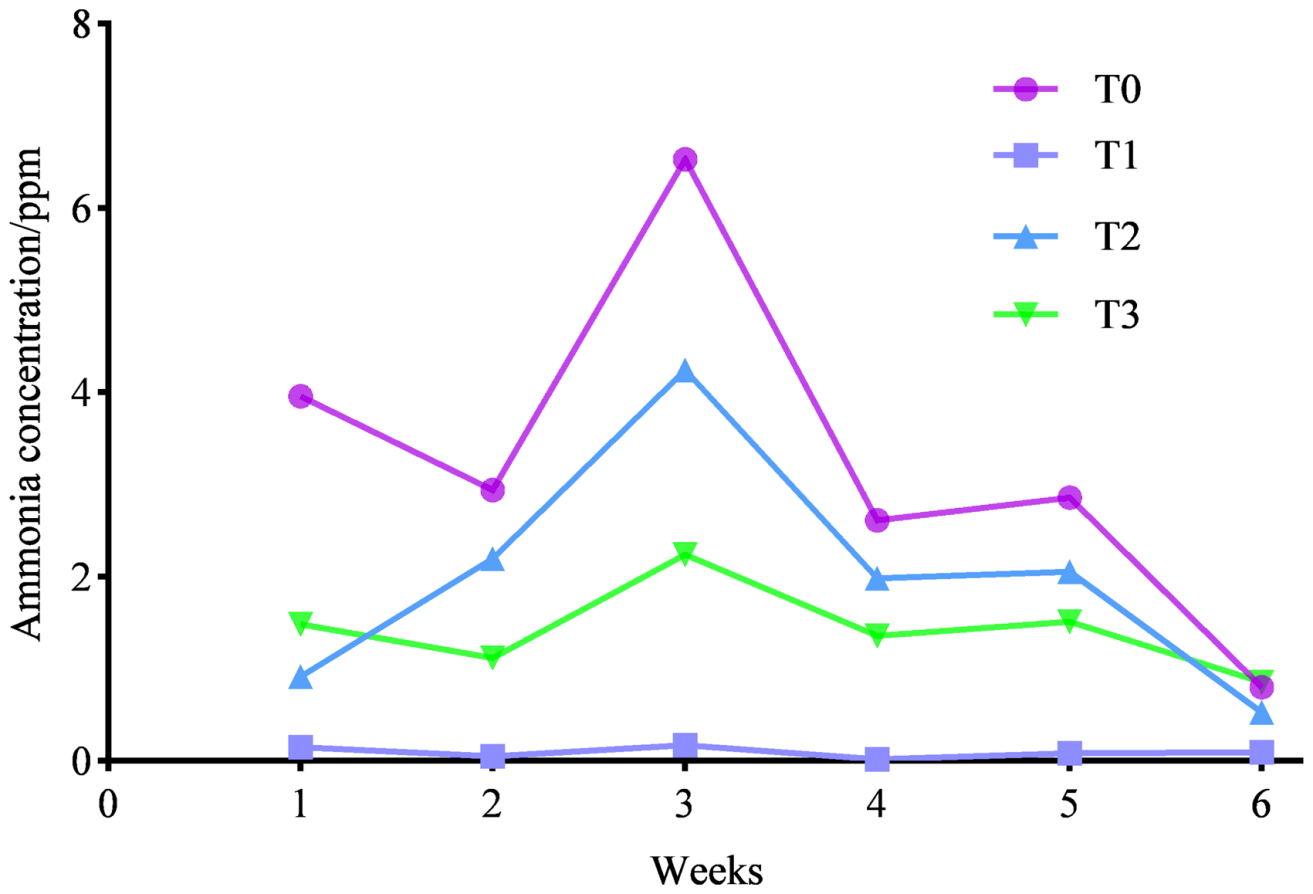
Effect of (TR)-fermented feed on the ammonia concentration in the chicken house.

As shown in Figure 2, we counted the ammonia emissions of each trial group and found that feeding (TR)-fermented feed decreased the ammonia emissions of birds (*p* < 0.05), and the ammonia emissions were T0 > T2 > T3 > T1 between each trial group.

### 3.6. Intestinal microflora

**Figure 3:**
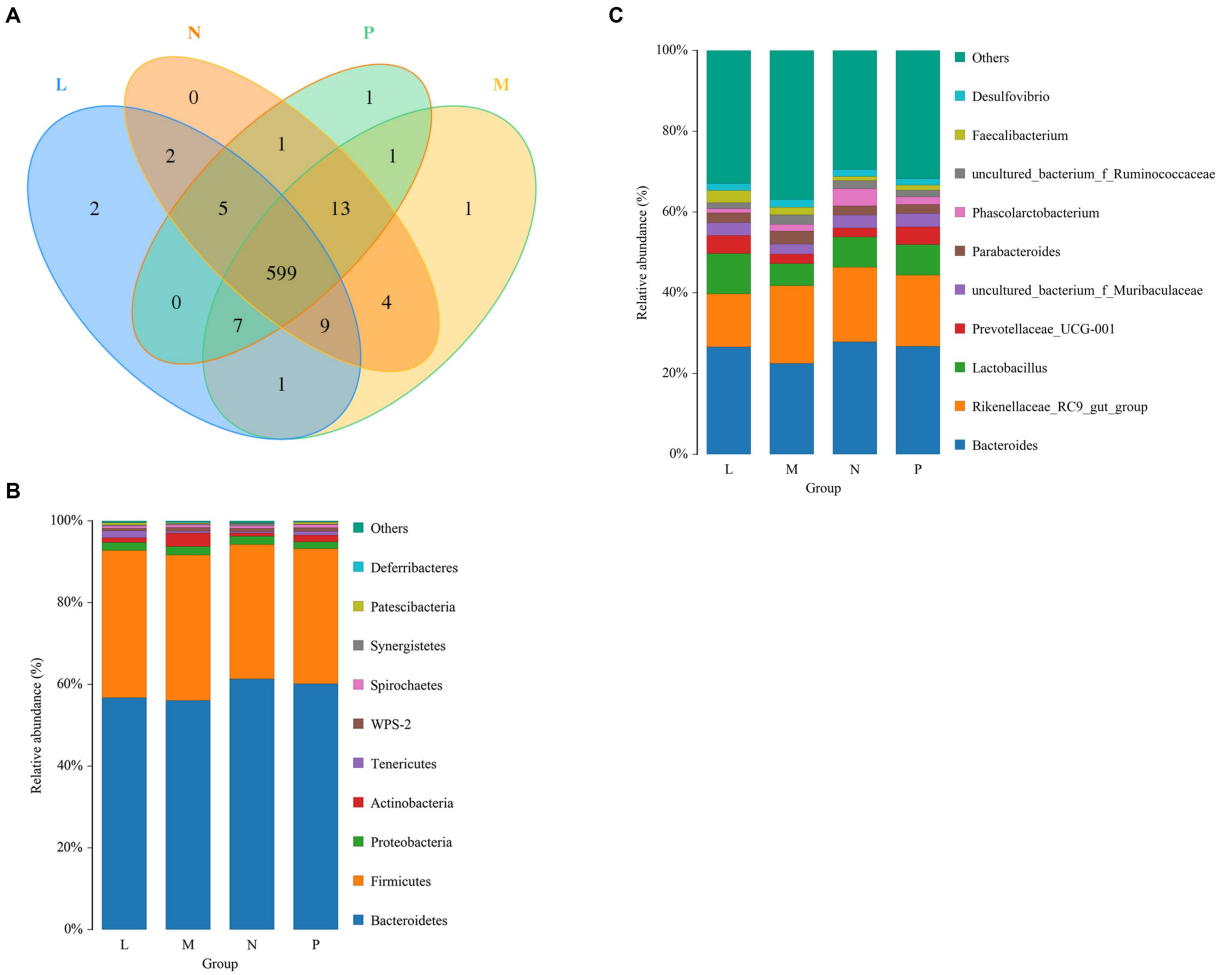
Laying hens’ bacterial community composition and Venn diagram on day 42. **(A)** The OTU Venn diagram. Phylum- or genus-level information on the composition of the bacteria in the cecum. “Other” includes all taxa with a relative abundance of less than 1%. P, control group; L, 1% (TR)-fermented feed; M, 3% (TR)-fermented feed; N, 5% (TR)-fermented feed. **(B,C)** The phylum and genus level compositions of the bacterial communities, respectively.

The cecal samples collected from laying hens yielded a total of 646 OTUs. Among the four groups, 599 OTUs were shared, while the remaining OTUs were specific to each group. Specifically, the L, M, N, and P groups had 2, 1, 0, and 1 specific OTUs, respectively (Figure 3A). Taxonomic compositions were examined at the phylum and genus levels to determine the variations brought about by (TR)-fermented feed in the cecal microbiota (Figures 3B,C). There were high relative abundances of 10 bacterial phyla. The two main phyla in the cecal bacterial community, *Bacteroidetes* and *Firmicutes*, were differentially abundant among groups, making up more than 55 and 33%, respectively. The relative abundance of *Tenericutes* increased significantly under the L group (*p* < 0.05).

*Bacteroides* was the dominant genus, followed by the *Lactobacillus* and *Rikenellaceae RC9* gut group. Lower abundances were 22.5% for *Bacteroides*, 14% for the *Rikenellaceae RC9* gut group, and over 8% for *Lactobacillus*. In addition, cecal bacteria with abundances greater than 1% were *Prevotellaceae UCG-001*, *uncultured bacterium f. Muribaculaceae*, *Parabacteroides*, *Phascolarctobacterium*, uncultured bacterium *f. Ruminococcaceae*, *Faecalibacterium*, and *Desulfovibrio*.

The M and N group decreased the abundances of *CHCKI001* and *Faecalibacterium* (*p* < 0.05; Table 7). The N group reduced the abundance of *GCA-900066575* (*p* < 0.01) and significantly improved the abundance of *Romboutsia* (*p* < 0.05). The L group significantly improved the abundances of *Flavonifractor*, *Pediococcus*, *Ruminiclostridium* 9 (*p* < 0.01), and *Ruminiclostridium* 5 (*p* < 0.05). Groups M and N significantly enhanced the abundance of *Fournierella* (*p* < 0.05). The M group significantly improved *Prevotellaceae* Ga6A1 (*p* < 0.01) and significantly reduced the abundance of *Sellimonas* (*p* < 0.05). Groups L and M significantly improved the abundance of *Ruminiclostridium* (*p* < 0.01) and significantly decreased the abundance of *Ruminococcaceae* UCG-014 (*p* < 0.05).

**Table 7:** Effect of different levels of fermented tea residue on the abundance of cecal bacterial genera (%).

Items	P	L	M	N	SEM	*p*-value
CHKCI001	0.26^a^	0.16^ab^	0.10^b^	0.05^b^	0.027	0.011
Flavonifractor	0.20^b^	0.34^a^	0.16^b^	0.23^b^	0.021	0.002
Fournierella	0.08^b^	0.26^a^	0.07^b^	0.14^ab^	0.11	0.034
GCA-900066575	0.29^ab^	0.38^a^	0.23^bc^	0.12^c^	0.029	0.001
Pediococcus	0.15^b^	0.66^a^	0.04^b^	0.26^b^	0.084	0.027
Prevotellaceae_Ga6A1_group	0.61^b^	0.18^b^	1.23^a^	0.22^b^	0.127	0.001
Romboutsia	0.35^b^	1.34^ab^	1.42^ab^	2.22^a^	0.238	0.028
Ruminiclostridium	0.21^b^	0.35^a^	0.35^a^	0.22^b^	0.022	0.004
Ruminiclostridium_5	0.29^b^	0.57^a^	0.30^b^	0.41^ab^	0.041	0.028
Ruminiclostridium_9	0.25^b^	0.59^a^	0.30^b^	0.27^b^	0.045	0.009
Ruminococcaceae_UCG-014	2.69^a^	1.10^b^	0.91^b^	1.69^ab^	0.238	0.015
Sellimonas	0.14^ab^	0.16^a^	0.09^c^	0.11^bc^	0.009	0.013
Faecalibacterium	2.99^a^	1.87^ab^	1.02^b^	1.28^b^	0.298	0.072

Values in the same row without or with the same letter superscripts indicate no significant difference (*p* > 0.05). Values with different small letter superscripts indicate a significant difference (*p* < 0.05).

**Figure 4:**
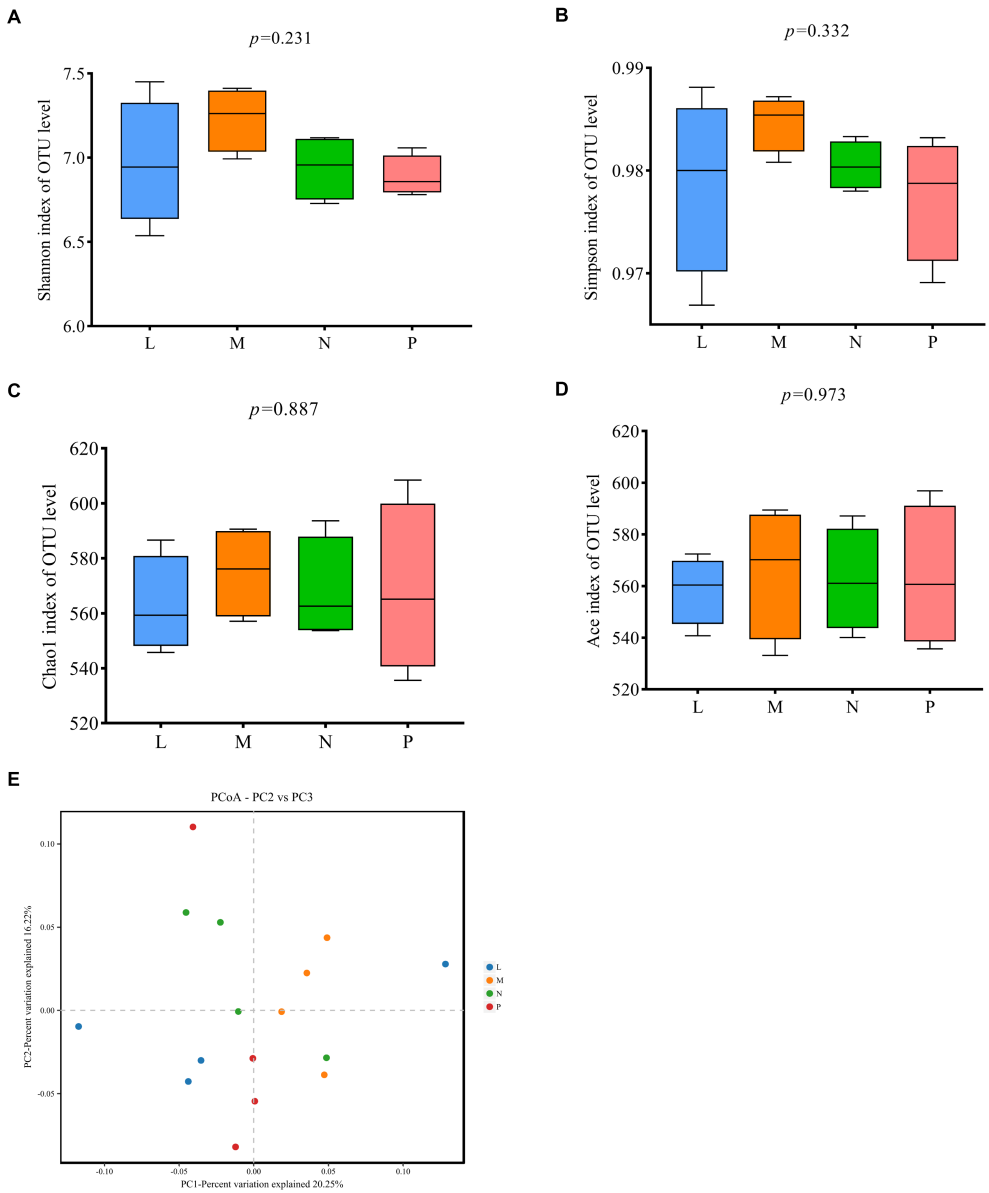
Analysis of the diversity of microbial communities between communities. **(A–D)** Alpha diversity analysis. **(E)** Principal coordinates analysis (PCoA) based on Bray-Curti’s distance. P, control group; L, 1% (TR)-fermented feed; M, 3% (TR)-fermented feed; N, 5% (TR)-fermented feed.

Figure 4 displays findings about the alpha and beta diversity of the cecal microbiota in laying hens. There were no discernible differences between the four groups for any of the alpha diversity indices, including Shannon, Simpson, Chao1 and Ace (Figures 4A–D). As shown in Figure 4E, principal coordinates analysis (PCoA) showed differences between the control group and the groups fed diets supplemented with 1, 3, or 5% (TR)-fermented feed, indicating differences in the cecal microflora between the control and experimental groups, but the differences were not significant.

## 4. Discussion

Tea residues are rich in tea polyphenols, these flavonoids can improve the secretion of estrogen and luteinizing hormone, thus improving the production performance of laying hens (28). There are a large number of probiotics in fermented feed, which can convert feed materials into microbial bacteriological proteins and bioactive small peptide amino acids and reduce anti-nutritional factors, thus promoting the utilization of nutrients in feed, and consequently improving feed conversion rate and animal performance (29). Some researches indicated that tea or fermented feed could enhance the production performance of laying hens by improving the digestion and absorption of feed (30–33). We found that feeding a certain amount of (TR)-fermented feed can improve laying rate and average egg weight and reduce feed-to-egg ratio (1 and 3%), but excessive addition does not have a positive effect (5%). We speculate that the reason may be related to the high content of polyphenolic composition in green tea residues, which can have a positive effect when added in moderation, but excessive addition will make its anti-nutritional effect significant, and its combination with animal oral salivary proteins will produce a bitter taste and reduce animal feed intake, thus reducing the production performance of hens.

We evaluated the quality of eggs mainly by albumen height, shell thickness, egg shape index, haugh unit and yolk color. The egg shape index is a parameter describing the shape of the egg, which has no effect on the nutritional value of food, but is related to the hatching and breaking rates which affect the breeding value (34, 35). Our study found that (TR)-fermented feed had no significant effect on the egg shape index. A higher haugh unit means that the albumen is more viscous and the egg quality is better (36). The color of the yolk is also an important indicator of the freshness of the egg. The deeper the yolk color, the better of the egg quality (37). In this experiment, the addition of a certain amount of (TR)-fermented feed can increase the haugh unit and yolk color of eggs. Eggshell thickness can directly affect the breakage rate of eggs. As fragile products, most egg losses during transportation and storage are caused by poor shell thickness. In this study, we found that the eggshell thickness of eggs increased by nearly half at higher additions (3, 5%). The thickness of the eggshell is related to the digestion and absorption of vitamins, calcium and phosphorus in the feed (38, 39), because the fermented feed contains a large number of vitamins and organic acids, which have substances that can promote the absorption of metal ions by the organism and thus increase the thickness of the eggshell (3, 40).

Eggs contain abundant protein, amino acids, fatty acids, vitamins, and other nutrients, and are an important source of nutrition for the human body. The amino acid and fatty acid contents of eggs are essential indices for assessing the egg’s nutritional value, as well as important flavor substances that greatly affect the taste and flavor of eggs. In this experiment, a total of 16 amino acids were detected, and after supplementation of (TR)-fermented feed, the TAA, EAA, NEAA, and DAA in eggs all increased, indicating that (TR)-fermented feed supplementation increased the content and freshness of amino acids in eggs, resulting in better quality. This is consistent with previous studies, which have shown that supplementing with tea polyphenols can increase the content of some amino acids in eggs (21). Eggs are rich in various fatty acids, and different fatty acids have different physiological effects on human health. Unsaturated fatty acids are vital essential fatty acids for the human body. Monounsaturated fatty acids have effects such as lowering blood sugar, reducing cholesterol, and regulating blood lipids. Polyunsaturated fatty acids have effects such as promoting brain development and preventing cardiovascular diseases (41, 42). Docosahexaenoic acid and eicosapentaenoic acid, two members of the omega-3 class of polyunsaturated fatty acids, are helpful for gastrointestinal cancer patients by enhancing immune function and help to lower triglycerides. Which is beneficial for heart health and helps improve conditions such as rheumatoid arthritis and other diseases (43). It was found that the addition of 0.1% green tea extract significantly increased the content of linoleic acid, arachidonic acid, docosahexaenoic acid and total polyunsaturated fatty acids in egg yolk (44). Supplementation of green tea by-products in the diet can increase the content of linolenic acid and docosahexaenoic acid in egg yolk (45). Our study found that the content of SFA, MUFA, PUFA, ω-3 polyunsaturated fatty acids, and ω-6 polyunsaturated fatty acids in eggs increased after supplementation with (TR)-fermented feed, indicating that supplementation with (TR)-fermented feed improved the fatty acid content in eggs of laying hens and greatly improved the quality of eggs.

In the antioxidant system, SOD, GSH-PX, and MDA metabolize free radicals *in vivo*, which is important for limiting oxidative damage in animals (46). Oxygen free radicals are biologically damaging macromolecules that are involved in the development of many diseases and the aging process of the body (47). The end product of lipid peroxidation brought on by oxygen radicals is M DA, and the amount of MDA in the body can indirectly indicate the amount of oxygen radicals present as well as the degree of cell damage (48). SOD is the first line of defense for scavenging free radicals in animals, which can rapidly convert superoxide anion radical O^2−^ into molecular oxygen and hydrogen peroxide (49). The main function of GSH-PX is to remove peroxide and H_2_O_2_ from the body (50). Our study found that feeding (TR)-fermented feed increased SOD and GSH-PX activity and decreased MDA content in laying hens. This indicates that feeding (TR)-fermented feed can reduce oxidative stress in laying hens. Some active substances in the (TR)-fermented feed and probiotics may increase the antioxidant capacity of the laying hens (51–53).

Ammonia is a harmful gas that is more harmful to laying hens. The concentration of ammonia is related to the protein levels in the diet and the digestibility of the poultry (54). Due to the short digestive tract of chickens, the digestion and absorption of feed are limited, and most of the nutrients are not digested and absorbed and excreted directly, especially proteins (55). Protein is decomposed by microbes under suitable conditions and releases a large amount of ammonia and other harmful gases, thus polluting the air of the chicken house (56). In this study, we found that feeding (TR)-fermented feed significantly reduced ammonia emissions in the laying hen house. According to similar studies, the addition of enzymes to the feed reduced nitrogen and ammonia emissions as well as the feeding of fermented *corn fructus* to broilers to reduce ammonia concentrations in broiler manure (57, 58). This is probably because the large molecules in the feed are broken down into small peptides and amino acids that are easily absorbed, reducing the amount of undigested protein in the intestine, and reducing the production of ammonia and toxic gases.

The intestinal flora of laying hens mainly colonizes the cecum, which can affect the health, immunity, digestive efficiency, and egg quality of laying hens (59). In this experiment, there was no significant difference in the relative abundance of *Bacteroides* and *Firmicutes* between the experimental and control groups, but adding 1% of (TR)-fermented feed had significantly more *Tenericutes* in the caecum of laying hens. It is possible that some probiotic bacteria in (TR)-fermented feed, such as *B. subtilis*, increased the abundance of *Tenericutes* (60). It was found that broilers fed fermented tea residue with probiotics or symbionts had a better feed conversion rate, but the relative abundances of *CHKCI001* and *Faecalibacterium* were lower than those of the control group (61). This is consistent with our M and N results. *Sellimonas* were negatively correlated with the average daily feed intake, feed conversion rate, and isobutyric acid levels (62). *Bacillus licheniformis* fermentation products increased the Coccidiococcus resistance index of broilers, but the relative abundance of *Sellimonas* in the cecum of broilers decreased, which was consistent with the results of the M group. *Pediococcus* is considered a good candidate probiotic for chickens (63). In this study, the relative abundance of *Pediococcus* was increased. A series of changes may be related to the addition of probiotics in the fermented tea residue feed. Probiotics and their metabolites act on the intestinal tract of laying hens and change the abundance of microbial flora.

Propionic acid, which is the primary byproduct of starch fermentation by starch breakdown bacteria, and Ruminococcaceae UCG-014 have a negative correlation with one another (64). The abundance of *Ruminococcaceae* UCG-014 of the L and M group was reduced. Adding (TR)-fermented feed to the diet may help to promote the digestion and absorption of feed for laying hens. Studies have found that *Romboutsia* is positively correlated with egg production by laying hens, and can be used to predict egg production (65). In our experiment, adding 5% (TR)-fermented feed significantly improved the relative abundance of *Romboutsia*. It is possible to increase the egg production of laying hens by adding (TR)-fermented feed. *Ruminiclostridium* is closely related to maintaining intestinal health. It can degrade cellulose and hemicellulose in plants and convert them into short-chain fatty acids, which are absorbed and used by the host (66). *Ruminiclostridium* 9 is directly proportional to the lipid metabolism capacity *in vivo* (65). *Ruminiclostridium* 5 has a positive effect on chicken body growth (67). The relative abundance of *Ruminiclostridium* 5 increased in group L. This may be related to the increased use of fermented tea residue feed by regulating the microflora. *Fournierella* can metabolize and produce acetic and propionic acids (68). Groups L and N had increased relative abundances of *Fournierella*, which may be related to the function of tea residue to enhance lipid metabolism.

## 5. Conclusion

Our findings revealed that the inclusion of a certain amount of (TR) fermented feed in the diet of laying hens has a positive effect on their production performance during the laying period. Additionally, it improves egg quality and serum antioxidant capacity, regulates the diversity of gut microbiota, and reduces ammonia emissions from poultry houses.

## Data availability statement

The original contributions presented in the study are publicly available. This data can be found at: https://www.ncbi.nlm.nih.gov/; PRJNA940200.

## Ethics statement

The animal study was reviewed and approved by the Leshan Academy of Agriculture Science.

## Author contributions

XC and XZ: conceptualization, methodology, formal analysis, investigation, and writing—original draft. SL: investigation, supervision, funding acquisition, and writing—review and editing. HZ and ZL: conceptualization, methodology, and formal analysis. All authors contributed to the study’s conception and design.

## Funding

This work was supported by Sichuan Science and Technology Program 2021JDRC0142.

## Conflict of interest

The authors declare that the research was conducted in the absence of any commercial or financial relationships that could be construed as a potential conflict of interest.

## Publisher’s note

All claims expressed in this article are solely those of the authors and do not necessarily represent those of their affiliated organizations, or those of the publisher, the editors and the reviewers. Any product that may be evaluated in this article, or claim that may be made by its manufacturer, is not guaranteed or endorsed by the publisher.
